# Structural and catalytic properties of copper silicate nanomaterials

**DOI:** 10.1038/s41598-020-57502-z

**Published:** 2020-01-16

**Authors:** Salem Bawaked, Katabathini Narasimharao

**Affiliations:** Department of Chemistry, Faculty of Science, King Abdulaziz University, P. O. Box 80203, Jeddah, 21589 Kingdom of Saudi Arabia

**Keywords:** Catalyst synthesis, Heterogeneous catalysis

## Abstract

Nanosized copper silicates with three different structural morphology (amorphous, nanotubes and MEL) were prepared using different synthesis methods. The physico-chemical properties of copper silicates were characterized by XRD, FT-IR, SEM, HRTEM, N_2_-physisorption, XPS and H_2_-TPR techniques. The results indicated that the preparation conditions affect reduction behavior and textural properties of nanosized copper silicates. Hydrothermal synthesis method yielded chrysocolla-like CuSiO_3_ nanotubes, which possessed high surface area and pore volume with easy reducibility. The catalytic performances of synthesized copper silicate nanostructures were evaluated for dehydrogenation of methanol. It was found that dehydrogenation activity is depended on the structural properties of copper silicates. Highest activity was observed for copper silicates with nanotube morphology. Catalytic dehydrogenation activity of copper silicates was also related to presence of more number of Cu-O-Si species, easy reducibility and Lewis acid centers. The CuSiO_3_ nanotubes sample also exhibited good stability under investigated reaction conditions that deactivation was not detected for 48 h.

## Introduction

Methanol (CH_3_OH) dehydrogenation is an important probe reaction that often utilized to investigate the chemical composition and surface characteristics of the catalysts^1^. The reaction can progress in several ways, resulting in either the formation of methyl formate (HCOOCH_3_) and formaldehyde (HCHO) or the decomposition of CH_3_OH into CO_x_ and H_2_. Interest in the latter process is associated with the remarkable progress in the H_2_ fuel cells research, which has taken place in recent decades^2^. Large scale production of HCHO is continuing due to the fact that it is widely used in manufacture of plastics and resins^3^. Methyl formate is also a highly reactive compound due to the concurrent existence of the ester group and labile hydrogen atom of the aldehyde group; this makes methyl formate as an advanced intermediate for the organic synthesis industry^4^. Methanol dehydrogenation to HCOOCH_3_ can be conducted either in the absence of oxygen (dehydrogenation) or presence of oxygen (oxidation).

The direct dehydrogenation is an industrially preferred process, because it yields gaseous hydrogen and methyl formate. However, the resulting yield is not more than 40%, since the equilibrium conversion is thermodynamically controlled. Among catalysts studied for the direct dehydrogenation, many catalysts usually containing copper as an active metal component^5^. There are many research reports existed in the literature with respect to development of stable heterogeneous catalysts for alcohol dehydrogenation focusing on the metal supported SiO_2_, Al_2_O_3_ and zeolites^6^. Copper supported catalysts showed an impressive performance in dehydrogenation of methanol. The performance of copper catalysts was found to be dependent on the catalyst synthesis conditions and physico-chemical properties, such as size of copper particles and specific surface area of catalyst^7^.

It was observed that methanol was selectively dehydrogenated to methyl formate over Cu/laponite and Cu/Na(Mg_2.5_Si_4_O_10_F_2_) clay catalysts, in contrast methanol dehydration to dimethyl ether was the predominant reaction over the Cu/saponite and Cu/montmorillonite catalysts^8^. It was also reported that the interlayer copper ions keep the initial oxidation state even in the reductive reaction conditions^9^. The low activity of copper supported clay catalysts is associated with unavailability of copper for methanol molecules because of its disposition in the interlayer space of the clay^10^. It was indicated in the literature that preliminary reduction treatment of copper catalysts (to convert oxidized Cu to Cu metal species) is necessary to obtain higher yields of methyl formate^11^. In contrast, some authors reported that not only metallic copper (Cu°) but oxidized copper species (Cu^2+^ and Cu^+^) also helpful for the formation of methyl formate in methanol dehydrogenation^12^. Therefore, the oxidation state of Cu, which is responsible for the catalytic performance in methanol dehydrogenation is still debatable.

Metal silicates are generally existed in different modes such as chains, sheets, rings and different framework structures^13–16^. The basic structural unit of metal silicates was found to be a tetrahedron structured anionic group with four negative charge, which is linked to each other^14^. Due to their unique structural and physico-chemical properties, the metal silicates were utilized in many applications such as catalysis, gas separation etc^15–17^. To meet industrial requirements, catalysts should possess a large and thermally stable active surface area. Thus, our aim is to develop porous nanostructured copper silicate catalysts, and investigate their promising application in methanol dehydrogenation. Recently, CuO-SiO_2_ nanocomposites were studied extensively due to the fact that SiO_2_ exhibits high thermal stability and it does not influence the physical nature of the CuO or nature of any other metal oxide, when it was used as a support^18^. In order to investigate the role of structural and textural properties of copper silicates nanomaterials in selectivity to methyl formate, three different nanostructured copper silicates with different structure (amorphous, MEL structure and nanotube) were synthesized. A careful structural and textural characterization of catalysts has been undertaken to determine the nature, as well as the role of the active sites responsible for dehydrogenation of methanol.

## Experimental Section

### Materials

All the reagents used in this work were obtained from Aldrich, U.K. They were used as received.

### Synthesis of copper silicate (CuSil) nanomaterials

#### Amorphous CuSil [CuSil-AMOR]

Sol-gel method was adapted to synthesize CuSil-AMOR sample^19^. Calculated amounts of tetraethyl orthosilicate and copper nitrate trihydrate were dissolved in ethanol and water (1:1 ratio) solvent in a round bottom flask and the contents were heated at 50 °C after adding a known amount of oxalic acid. The stirring was continued while heating is continued to remove the solvent from the clear solution and was allowed to form a gel. The obtained gel was dried in a conventional electric oven at 120 °C for 12 hours. The collected sample was then powdered and calcined in a muffle furnace under flowing air at 500 °C for 5 hours.

#### CuSil nanotubes [CuSil-NT]

The CuSil-NT sample was synthesized by using hydrothermal method described in the literature^20^. A known amount of copper nitrate trihydrate (0.5 g) was dissolved in 5 mL of mixed solvent (distilled H_2_O and ethanol in the ratio of 1:4). To this 5 mL of 0.5 M sodium silicate solution was added to obtain light blue precipitate and it was poured into a Teflon lined stainless steel pressure vessel and the contents were subjected to hydrothermal treatment at 200 °C for 48 hours. After cooling the vessel, the contents were filtered and washed with distilled H_2_O and ethanol. Finally, the material was dried at 100 °C for 2 hours and then calcined at 500 °C for 5 hours.

#### MEL structured CuSil [CuSil-MEL]

The synthesis of CuSil-MEL material was carried out using hydrothermal method^21^. Typically, stoichiometric quantities (1 CuO: 90 SiO_2_: 9 TBA_2_O: 6.5 Na_2_O: 1055 H_2_O) of copper nitrate, tetraethyl orthosilicate and sodium hydroxide were mixed in ethanol-water solvent and stirred for 30 min. To this solution, calculated amount of tetrabutyl ammonium hydroxide (TBAOH) was added under constant stirring. The temperature of the mixture was adjusted to 0 °C by using placing the flask in ice bath (kept it for 1 h), and then the flask was moved to hot water bath to maintain the temperature of the flask at 50 °C for 3 more hours to obtain a gel. The obtained gel was sealed in a Teflon-lined stainless-steel pressure vessel and it was thermally treated at 180 °C for 7 days. After the hydrothermal treatment, the autoclave was quenched with cold water, and the formed product was separated by centrifugation and it was washed with distilled water. The washed material was dried at 100 °C for 12 h and then calcined at 500 °C for 5 h under the flow of air.

### Characterization of synthesized CuSil nanomaterials

The elemental composition of the synthesized materials was determined by using ICP-AES, Optima 7300DV (Perkin-Elmer) instrument. Powder X-ray diffraction measurements of CuSil materials were carried out using Bruker D8 advance target diffractometer with Cu Kα (λ = 1.5405 Å) radiation. The detection of crystalline phases presented in the prepared materials was accomplished by comparing XRD patterns with JCPDS files. The FT-IR spectra of fresh and pyridine adsorbed CuSil materials were obtained using Bruker D70 spectrometer. The scanning electron microscopy images of the samples were obtained over the JEOL JSM840A instrument. The powder sample was attached to an aluminum block using double sided carbon tape and the images were obtained at different magnifications. TEM images of synthesized materials were obtained using JEOL 2010, 200 kV transmission electron microscope. The nitrogen adsorption-desorption isotherms for all the materials were obtained using NOVA 3200e (Quantachrome, USA) automated gas sorption system. The specific surface area (S_BET_) of the materials were determined using Brunauer-Emmett-Teller (BET) equation. The pore size distribution patterns of the samples were obtained using NLDFT method. The X-ray photoelectron spectral data for all the samples were obtained using SPECS GmbH XPS instrument as described in our previous publication^22^. Hydrogen-temperature programed reduction (H_2_-TPR) measurements were carried out using Quantachrome CHEMBET 3000 apparatus equipped with thermal conductivity detector.

### Catalytic dehydrogenation of methanol (CH_3_OH)

The catalytic activity of the synthesized CuSil samples for oxygen free vapor phase dehydrogenation of CH_3_OH was determined using fixed bed micro reactor. Helium gas saturated with methanol vapor to 10 vol % was used as the reaction mixture. The rate of the supply of the reaction mixture was 15 L h^−1^. The catalyst was loaded into the reactor in the volume of 2 cm^3^. The contact time of the reaction mixture with the catalyst was 0.4 s. The catalytic runs were carried out within a temperature range of 250–500 °C. The catalyst was kept for 30 min at each temperature in order to reach the steady state. The analysis of the composition of the reaction mixture at the inlet and outlet of the reactor was performed using gas chromatography. In order to separate the components of the mixture, Porapak T type chromatographic column was used. In order to determine the stability of the samples, the temperature of the reactor was increased to the reaction temperature (the temperature of the maximum yield of the target product on this catalyst) for 2 h. The time of testing of the samples at the steady state temperature was 48 h.

## Results and Discussion

**Figure 1:**
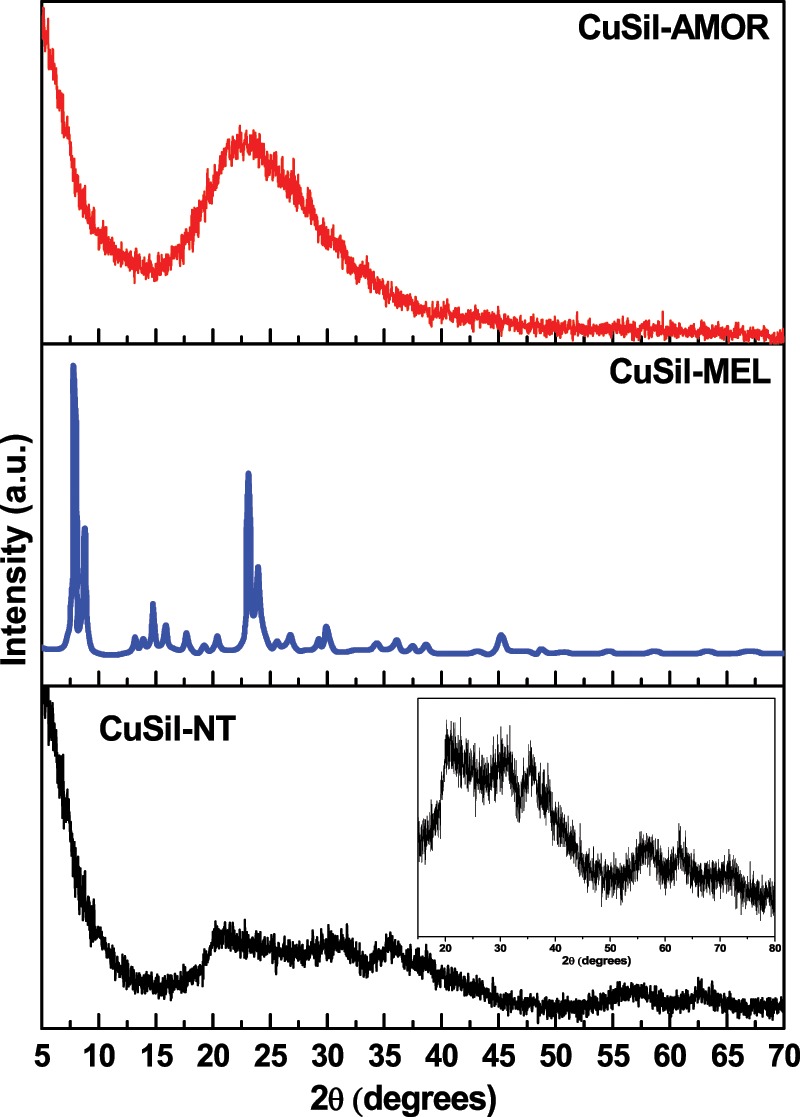
XRD patterns of calcined CuSil samples.

The XRD patterns of calcined CuSil samples are presented in Fig. 1. The XRD pattern of CuSil-NT sample shows broad diffraction peaks, which are in agreement with the XRD pattern of copper silicate hydrate phase [CuSiO_3_·xH_2_O, JCPDS No. 00–003–1152]. The XRD pattern of the CuSil-AMOR sample shows a broad reflection centered at 2θ = 22° similar as amorphous silica, revealing that this sample possessed amorphous copper silicate structure. In contrast, CuSil-MEL is highly crystalline and exhibited characteristic reflections due to presence of MEL structure^23^. No additional diffraction peaks due to copper oxides were observed in the three samples, indicating the effectiveness of the adopted synthesis methods to obtain the pure nanosized copper silicate materials.

**Figure 2:**
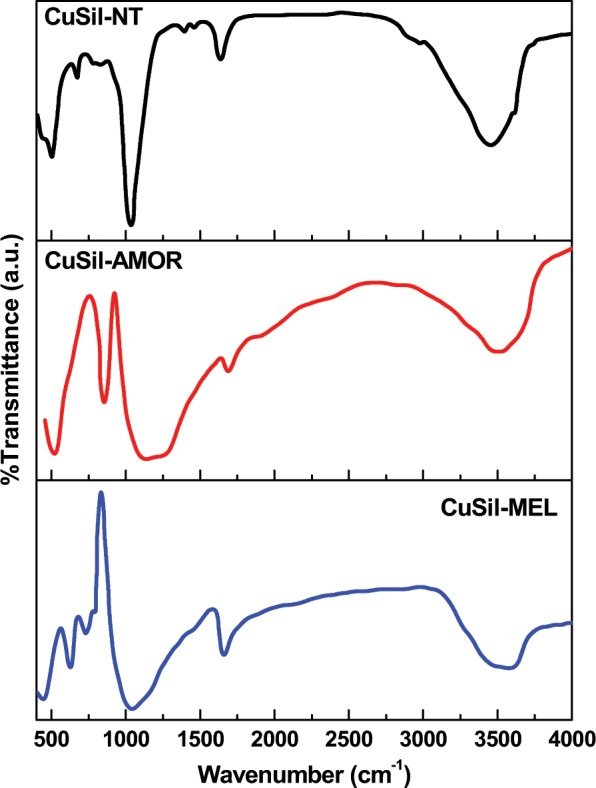
FTIR spectra of calcined CuSil samples.

The FT-IR spectrum for CuSil-NT sample (Fig. 2) displayed two bands at 3620 cm^−1^ and 673 cm^−1^, which could be attributed to the stretching and bending vibrations of hydroxyl groups attached to the copper atoms. The two bands appeared at 3450 cm^−1^ and 1630 cm^−1^ can be assigned to stretching and bending vibrations of adsorbed water molecules. The sharp band observed at 500 cm^−1^ is due to the Cu-O-Si bending vibration and the most intense band at 1035 cm^−1^ could be ascribed to Si-O stretching vibration of silicate tetrahedrons^24^. The remaining two bands at 825 cm^−1^ and 775 cm^−1^ could be assigned to the stretching vibrations of silicate chains^24^. The FT-IR spectrum of CuSil-MEL sample showed absorption bands at 460, 550 and 790 cm^−1^, which could be attributed to the presence of MEL structure in the synthesized sample^25^. The band at 460 cm^−1^ can be assigned to bending vibration of O-Si-O bonds and the bands at 550 and 790 cm^−1^ are due to the symmetrical stretching vibration of tetrahedral linkage and double ring vibrations respectively^26^.

The band appeared at 1650 cm^−1^ is due to the H-O-H bending vibration of H_2_O molecules bound to MEL structure. The FT-IR spectrum of Cu-MEL sample also exhibited a band at 3550 cm^−1^, which is due to stretching vibration of physically adsorbed H_2_O molecules to the MEL structure. FT-IR spectrum of CuSil-AMOR sample exhibited a band due to *δ*(-OH) at 670 cm^−1^ and a shoulder due to *ν*(Si-O) at 1042 cm^−1^ and also an asymmetric stretching Si-O vibration due to SiO_2_ at 1100 cm^−1^ ^27^. It is important to note that the characteristic absorption around 630 cm^−1^ for CuO was not observed in all samples, which indicates the absence of CuO particles in the synthesized samples^28^.

**Figure 3:**
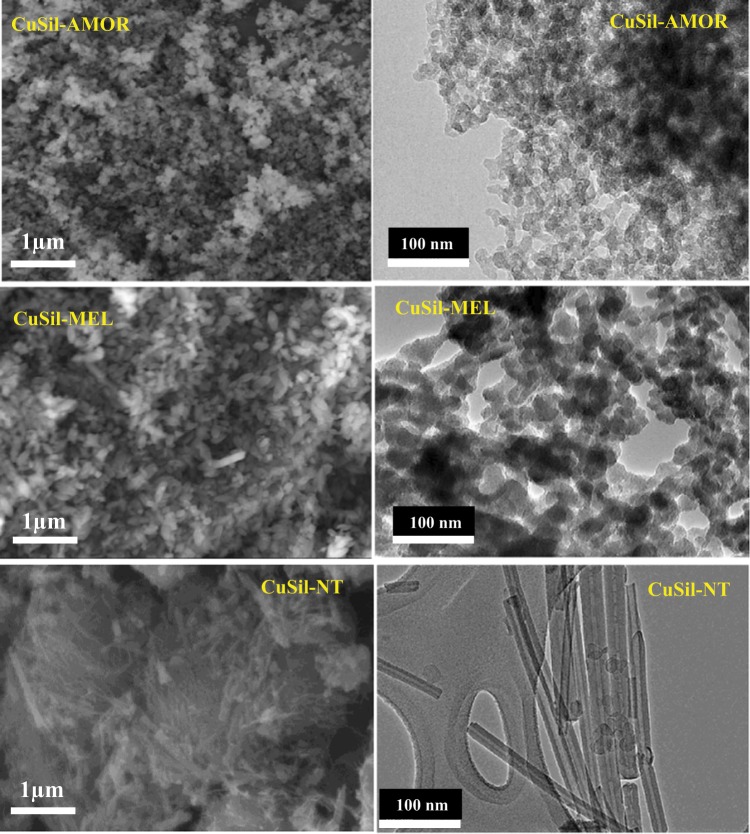
SEM and TEM images of calcined CuSil samples.

The SEM and TEM images of synthesized CuSil samples are shown in Fig. 3. The SEM micrographs reveal that the three samples possessed crystallites with different type of morphology. The morphology of the samples is different due to the fact that three different preparation procedures were adopted to synthesize the samples. The size of the particles could be accurately calculated from the TEM analysis. The CuSil-AMOR sample possessed spherical and worm shaped particles of size in between 15–25 nm. Figure 3 shows that the CuSil-MEL sample composed of mainly spherical particles with diameters of 25 nm, and some of the particles in this sample were aggregated as spheroids, packed together in a random manner. The CuSil-NT sample is consisted of large number of accumulated nanotubes as shown in Fig. 3. Most of the nanotubes positioned horizontally on surface of the sample and the diameter of all nanotubes have narrow size distribution. A representative TEM image of the CuSil-NT sample is shown in the figure, in which the diameter and length are about 20 nm and 300 nm, respectively.

**Figure 4:**
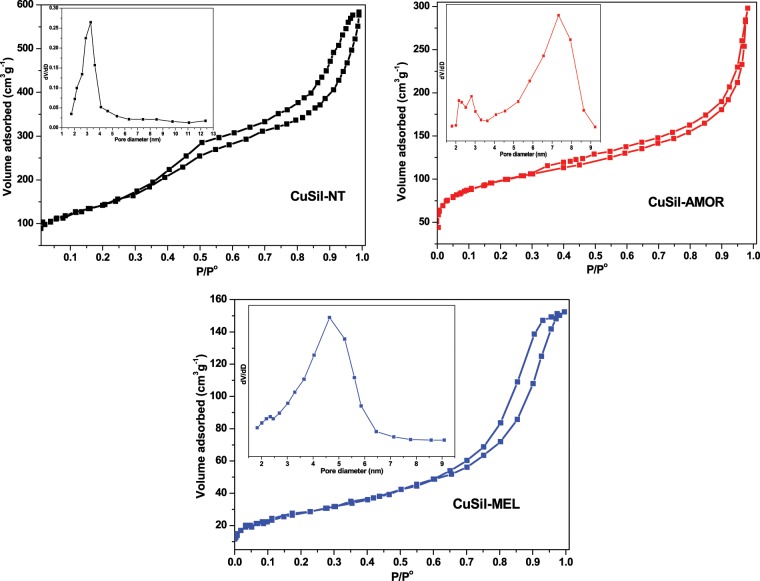
N_2_ adsorption-desorption isotherms and pore size distribution patterns (inset) of CuSil samples.

The N_2_ adsorption-desorption isotherms and pore size distribution patterns (in the inset) of calcined CuSil samples are shown in Fig. 4. The synthesized CuSil materials exhibited isotherms of type-IV with H2-type hysteresis loops, which is an indication that the materials possessed meso pore structure^29^. The slight slope in the adsorption-desorption isotherms in the low relative pressures revealing that these samples possessed relatively small amount of micro size pores, except the slope is relatively higher in case of CuSil-AMOR sample, which was also clearly observed in BJH pore size distribution patterns of the samples. The major textural characteristics of the synthesized materials are presented in Table 1.

**Table 1:** Textural properties of the CuSil samples.

Catalyst	BET surface area (m^2^g^−1^)	Pore volume (cm^3^g^−1^)	Pore diameter (nm)
CuSil-AMOR	410	0.650	7.6
CuSil-MEL	552	2.523	4.5
CuSil-NT	365	0.592	3.2

The CuSil-NT sample possessed the BET surface area and pore volume of 365 m^2^g^−1^ and 0.592 cm^3^g^−1^, respectively. This sample exhibited narrow pore size distribution centered at 3.2 nm, which is lower than the other two samples. The CuSil-MEL sample exhibited high large pore volume, around 2.523 cm^−3^g^−1^, and high specific surface area of 552 m^2^g^−1^ majorly due to the fact that this sample possessed intracrystalline mesopores resulting the high porosity.

**Figure 5:**
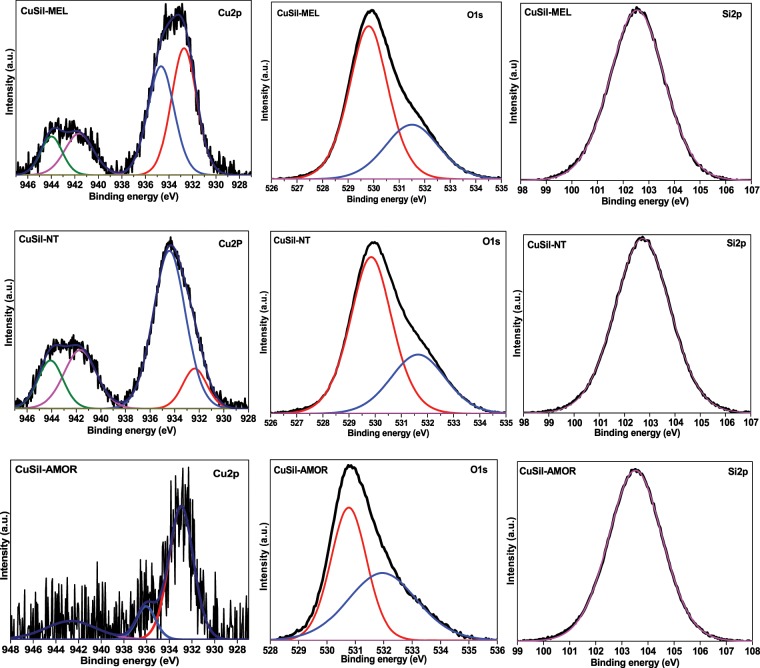
Deconvoluted XP spectra of CuSil samples.

The XPS analysis was performed to understand the electronic state of Cu, Si, and O elements in the thermally treated CuSil samples. The deconvoluted X-ray photoelectron spectra of the materials are shown in Fig. 5. It was previously indicated that the binding energy (BE) for Cu *2p*_*3/2*_ photoelectron core level in Cu^+^ and Cu^2+^ species are 932.4 eV and 933.5 eV respectively^30^. It was also reported that materials, which contained Cu^2+^ species exhibit strong satellite XP peaks, with BE at 6 to 10 eV above the main core level peaks and these satellite peaks does not appear in materials containing Cu^+^ species^31^. The three CuSil samples exhibited two XP peaks corresponding to Cu species; the first peak with a Cu *2p*_*3/2*_ BE of 933.2 eV and the second one at 935.4 eV. The peak at 933.2 eV is consistent with Cu^2+^ species. The peak at 935.4 eV could be attributed to the Cu species in Cu-O-Si network. Mosser *et al*.^32^ also observed BE of 935 eV for Cu species in natural and synthetic copper silicate minerals. The quantification of species corresponding to different surface species was performed and the obtained results are tabulated in Table 2. It is clear that CuSil-MEL and CuSil-NT samples possessed more surface Cu-O-Si species than CuSil-AMOR sample.

**Table 2:** Bulk & surface elemental composition and acidity of CuSil samples.

Catalyst	Acidity of the catalysts		Bulk composition (mass%, ICP-AES)			Surface composition (mass%, XPS analysis)				
	Brønsted acid sites	Lewis acid sites	Cu	O	Si	Cu		O		Si
						Cu^2+^	Cu-O-Si	O_lattice_	O_interactive_	
CuSil-AMOR	15.6	20.1	29.5	40.3	30.2	25.2	6.3	31.0	8.8	28.7
CuSil-MEL	18.4	26.6	28.3	39.2	32.5	15	18.0	27.4	10.0	29.6
CuSil-NT	23.3	35.9	29.8	39.6	30.6	6.9	25.4	28.0	11.3	28.4

The observed O *1s* XP spectra in CuSil samples are broad and the deconvolution of the peaks yielded in two different types of O *1s* species with BE in the range of 529.8–530.7 eV and 531.5–532 eV. The O*1s* peak appeared at the range of 529.8–530.7 eV could be assigned to oxygen in Cu-Si-O species and the peak at the range of 531.5–532 eV is consistent with core level oxygen in Cu and Si oxides^33^. The Si *2p* peaks for the three CuSil samples also included in the figure. The observed binding energy of 102.9 eV for the Si *2p* in CuSil-NT and CuSil-MEL samples suggests that surface Si species are in the silicate structure as the BE is higher for SiO_2_ (103.5 eV)^34^. However, CuSil-AMOR sample clearly exhibited the Si *2p* peak at 103.5 eV corresponding to silicate species, the observed shift in the binding energy could be due to presence of more surface SiO_2_ species in this sample.

**Figure 6:**
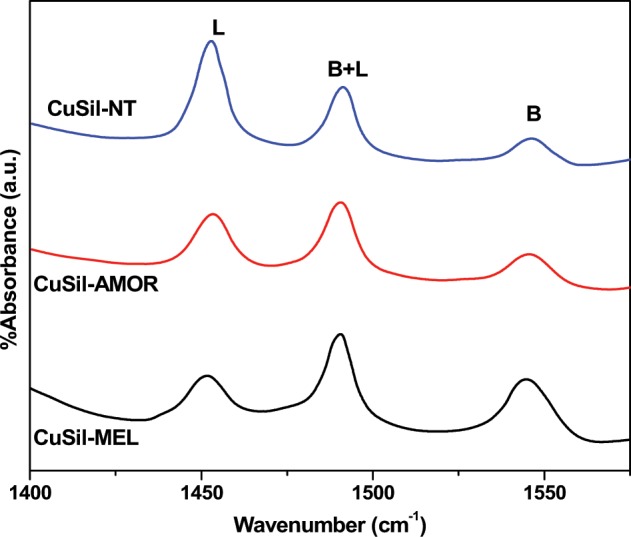
FTIR spectra of CuSil samples after pyridine adsorption.

The acidic properties of the CuSil samples are investigated using the FT-IR spectra after pyridine adsorption. FT-IR spectra of pyridine adsorbed CuSil materials are presented in Fig. 6. It was reported that two bands at 1450 cm^−1^ and 1610 cm^−1^ corresponding to the pyridine molecules interacted with Lewis (L) acid sites and the bands at 1545 cm^−1^ and 1635 cm^−1^ could be attributed to the pyridine interacted with Brønsted (B) acid sites^35^. The bands around 1490 cm^−1^ represents the pyridine molecules bound to both L and B acid sites^36^. The synthesized CuSil samples exhibited bands due to both Lewis and Brønsted acid sites, and the quantification of the amount of the Lewis and Brønsted acid sites was performed using reported procedure^37^. The results are presented in the Table 2. It is clear that CuSil-NT sample possessed higher amount of both Lewis and Brønsted acid sites than the other two CuSil samples. It is majorly due to the fact that CuSil-NT material possessed more number of surface silanol groups and Cu^2+^ species than the other two samples, which are related to the Brønsted and Lewis acid sites^38^.

**Figure 7:**
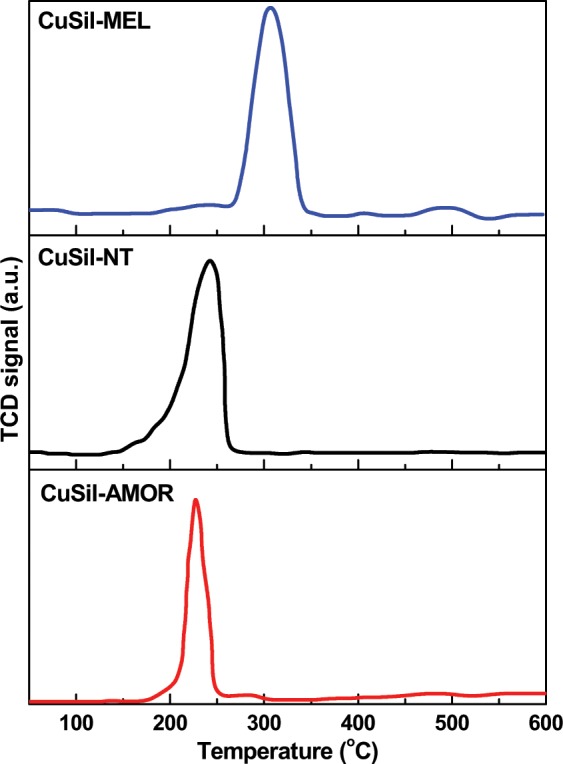
H_2_-TPR patterns of CuSil samples.

The H_2_-temperature programed reduction (H_2_-TPR) patterns for the three calcined CuSil materials are presented in Fig. 7. The H_2_-TPR patterns of the materials indicated that the three CuSil samples have one major reduction peak, which could be assigned to the reduction of Cu^2+^ species to metallic copper (Cu°). However, the reduction peak temperature varied from 240 to 320 °C, in which both CuSil-AMOR CuSil-NT samples have the lowest reduction peak temperature, while CuSil-MEL sample has a reducing peak temperature about 320 °C. This observation is indicating that CuSil-AMOR and CuSil-NT samples are easily reducible than CuSil-MEL sample. The observations from the H_2_-TPR results reveals that the copper silicate structure have an influence on the reducibility of Cu species.

### Catalytic dehydrogenation of methanol

All the catalysts were tested for dehydrogenation of methanol; methyl formate and dimethyl ether were observed as major reaction products and a small amount of methane was also observed under the studied reaction conditions. All the results presented in this work were obtained under steady-state conditions. Initially, influence of reaction temperature over the methanol conversion and selectivity to methyl formate over the CuSil catalysts was studied (Table 3) at GHSV of 250 h^−1^. The bare SiO_2_ and CuO samples also tested to compare their catalytic activities with the CuSil samples. It was observed that the bare SiO_2_ and CuO samples exhibited poor activity. It can be observed that SiO_2_ and CuO are highly selective towards methyl formate, but the conversion of methanol is very low, as the yields are not higher than 4%. Consequently, the catalyst activity is simply related to formation of copper silicate structures. The synthesized CuSil samples are active and selective towards methyl formate formation.

**Table 3:** Catalytic methanol dehydrogenation over investigated samples.

Catalyst	Reaction temperature (°C)	Methanol conversion (%)	Selectivity to methyl formate (%)	Activity (µmol g^−1^ min^−1^)
CuSil-AMOR	350	18.1	72.1	0.89
	400	32.3	85.2	1.52
	450	45.4	70.8	2.32
	500	56.7	64.5	2.98
CuSil-MEL	350	20.2	74.2	1.02
	400	35.3	87.3	1.81
	450	47.8	73.5	2.46
	500	58.9	67.9	3.03
CuSil-NT	350	22.7	92.1	1.23
	400	39.8	93.2	1.94
	450	55.2	74.1	2.62
	500	69.3	68	3.31
CuO	350	2.6	90	0.11
	400	4.7	85.2	0.19
	450	8.9	81.4	0.26
	500	11.6	78.3	0.3
SiO_2_	350	1.1	92.1	0.06
	400	2.9	90.5	0.13
	450	5.2	84.3	0.2
	500	7.9	80.5	0.25

GHSV = 1000 h^−1^.

The CuSil-NT sample exhibited higher activity compared to CuSil-MEL and CuSil-AMOR samples. Although, the conversion of methanol was increased with increase of reaction temperature in the CuSil samples and the selectivity to methyl formate decreased with increase of reaction temperature. This is possibly be due to the fact that at higher reaction temperatures, the thermal decomposition or decarbonylation of methyl formate could occur on surface of the catalyst and also a non-catalytic thermal decomposition of methanol is also possible at very high reaction temperatures [reactions (2) to (4)]^39^.

**Figure 8:**
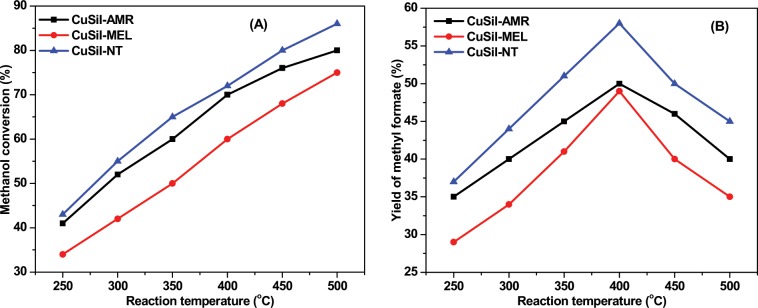
The temperature dependence on (**A**) methanol conversion (**B**) yield of methyl formate over CuSil samples (GHSV = 250 h^−1^).

Figure 8 illustrates the temperature dependence on methanol conversion and methyl formate yield for CuSil samples in a wide temperature range (250–500 °C). All samples were catalytically active even at low reaction temperatures (250 °C) and the conversion increased almost linearly with increase of reaction temperature. At low reaction temperatures (250 °C to 400 °C), high levels of methyl formate yields were observed mainly due to high selectivity to dehydrogenation product methyl formate. As a result of temperature increase (450 °C and 500 °C), an intensification of the reactions (2) to (4) occurred leading to significant drop in methyl formate yield. The highest methyl formate yield (57% at 400 °C) was obtained for CuSil-NT sample. The obtained results clearly indicating that presence of copper active sites is required as bare oxides showed no activity and high reaction temperatures affect negatively on the selectivity and yield of methyl formate.

1 $$2{{\rm{CH}}}_{3}{\rm{OH}}\leftrightarrow {{\rm{HCOOCH}}}_{3}+{{\rm{H}}}_{2}$$

2 $${{\rm{HCOOCH}}}_{3}\leftrightarrow 2{\rm{CO}}+2{{\rm{H}}}_{2}$$

3 $${{\rm{HCOOCH}}}_{3}\leftrightarrow {\rm{CO}}+{{\rm{CH}}}_{3}{\rm{OH}}$$

4 $${{\rm{HCOOCH}}}_{3}\leftrightarrow {{\rm{CO}}}_{2}+{{\rm{CH}}}_{4}$$

**Figure 9:**
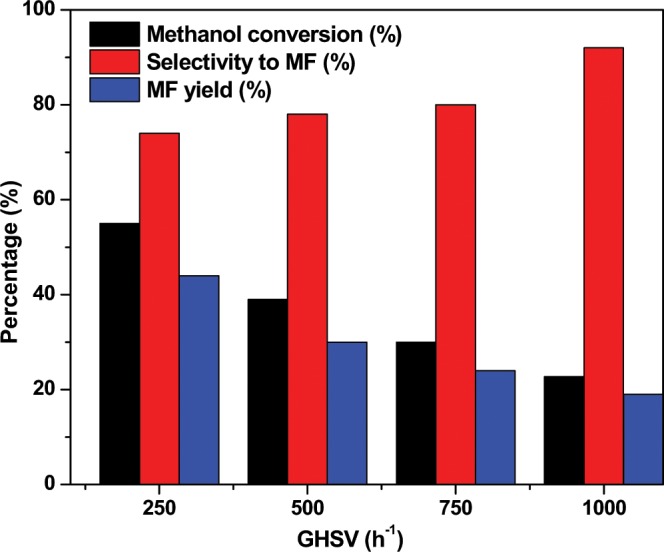
The influence of GHSV on methanol dehydrogenation over CuSil-NT sample.

The influence of the gas hourly space velocity (GHSV) on the catalytic methanol dehydrogenation activity for the synthesized CuSil samples was also studied. Figure 9 presents the conversion of methanol, selectivity to methyl formate observed at various GHSV values in case of CuSil-NT catalyst at 300 °C. It is clear that the catalyst offered high methanol conversion and low methyl formate selectivity at low GHSV values; probably, degradation of methyl formate taking place on the catalyst surface due to high residence time. However, no methane was detected in the reaction products, due to the fact that degradation of methyl formate [reactions (2) and (3)] yields CO and H_2_. Based on the observed results, and in order to obtain the high methyl formate yields, a GHSV of 250 h^−1^ was selected for other catalytic tests.

The reaction kinetic study was carried out assuming that the reactions (1) and (2) are taking place over the CuSil-NT catalyst at low reaction temperature. Rate constants for reactions (1) and (2) were determined from the obtained activity data such as conversion of methanol, selectivity and yield of methyl formate. The estimation of kinetic parameters was carried out by following the procedures reported by Shelepova *et al*.^40^. The activation energy and pre-exponential factor for synthesized catalysts was performed at two different reaction temperatures (250 and 300 °C). The average error have not exceeded 5% in all catalysts (Table 4). The rate constants at 250 °C and 300 °C for CuSil-NT sample are 6.5 × 10^3^ M^−1^s^−1^ and 1.5 × 10^3^ M^−1^s^−1^ respectively. For this catalyst, the observed pre-exponential factor and activation energy are 9.1 × 10^7^ M^−1^s^−1^ and 34.4 kJ mol^−1^ for methanol dehydrogenation. However, these values are around 5.6 × 10^8^ s^−1^ and 52.4 kJ mol^−1^ for methyl formate decomposition. It was observed that the rate constant k_1_ increased with increase of reaction temperature in case of three CuSil samples.

**Table 4:** Reaction kinetic studies of over CuSil samples.

Catalyst	Reaction temperature (^o^C)	K_1_ (M^−1^s^−1^)	K_10_ (M^−1^s^−1^)	K_20_ (s^−1^)	E_a1_ (kJmol^−1^)	E_a2_ (kJmol^−1^)
CuSil-AMOR	250	5.3 × 10^2^	3.7 × 10^6^	7.2 × 10^6^	52.3	71.1
	300	0.7 × 10^3^	2.3 × 10^7^	4.6 × 10^7^	44.7	54.7
CuSil-MEL	250	6.0 × 10^2^	7.2 × 10^6^	2.4 × 10^7^	46.1	79.2
	300	0.9 × 10^3^	4.1 × 10^7^	3.9 × 10^7^	38.5	51.4
CuSil-NT	250	6.5 × 10^3^	9.1 × 10^7^	5.6 × 10^8^	34.4	52.4
	300	1.5 × 10^3^	7.9 × 10^8^	2.1 × 10^9^	29.8	46.1

It was previously reported that when copper was supported on weakly acidic or amphoteric support, highest methyl formate yields were observed. On other hand, when the copper was supported on basic support, both methyl formate selectivity and methanol conversion decreased^5^. It was also observed that copper crystal size have not shown any significant influence on the catalytic dehydrogenation performance in copper-silica catalysts^41^. However, the observed results indicating that crystal structure and morphology of the copper silicates played an important role in dehydrogenation of methanol reaction.

**Figure 10:**
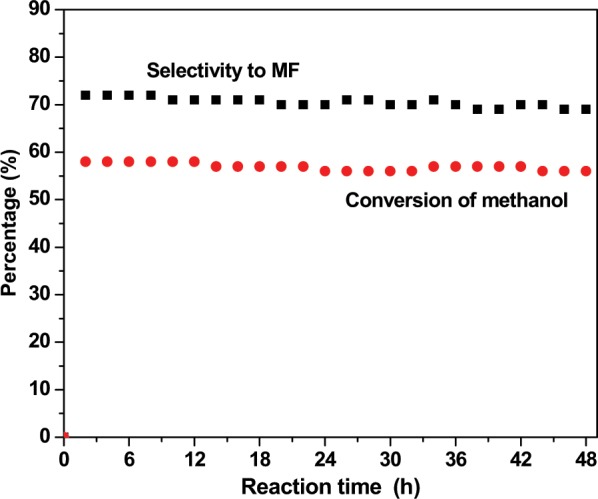
Time on stream analysis of CuSil-NT catalyst [GHSV = 250 h^−1^, temp: 400 °C].

It is also known that reduced copper species but not the Cu^2+^ are the active species for methanol dehydrogenation^42^. The chemical state of copper in the synthesized CuSil samples determined by XPS analysis indicated that CuSil-NT sample possessed more number of surface Cu species with oxidation state lower than 2 + . In the case of CuSil-MEL and CuSil-AMOR, it seems that copper particles are interacting with surface hydroxyl groups (as FTIR results showed low intense bands due to -OH groups), thus accomplishing copper stabilization at a higher oxidation state. The most active CuSil-NT sample was selected to study the durability of copper silicate catalyst. Figure 10 presents the time on stream behavior of the sample. The catalyst possessed its initial activity even after 48 hours of reaction, revealing the stability of the CuSil-NT sample. The enhanced catalytic dehydrogenation activity of CuSil-NT catalyst could be due to the fact that CuSil-NT possessed greater number of surface Lewis acid sites and interactive [Cu-O-Si] species, which are easily reducible.

## Conclusions

Copper silicate materials with amorphous, nanotube and MEL structures were synthesized by adapting various synthesis methods. A thorough characterization of synthesized materials was performed using powder XRD, FT-IR, SEM, HRTEM, N_2_-physisorption, XPS and H_2_-TPR techniques. The synthesized copper silicate nanotubes sample possessed high surface area, pore volume and easy reducibility compared to other two samples. The catalytic performance of copper silicate nanostructures were evaluated in vapor-phase dehydrogenation of methanol. It was found that catalytic dehydrogenation activity is depended strongly on the structural properties of copper silicates. The enhanced catalytic dehydrogenation activity of CuSil-NT catalyst could be due to the fact that CuSil-NT possessed greater number of surface Lewis acid sites and interactive species, which are easily reducible. The copper silicate catalysts also showed good stability under reaction conditions without any considerable catalyst deactivation.

### Ethics

This article does not present research with ethical considerations.

## References

[1] Badlani M, Wachs IE (2001). Methanol: A “Smart” Chemical Probe Molecule. Catal. Letts..

[2] Jiang R (2011). Methanol dehydrogenation on Rh (111): A density functional and microkinetic modeling study. J. Mol. Catal. A: Chem..

[3] Geyer R, Jambeck JR, Law KL (2017). Production, use, and fate of all plastics ever made. Sci. Adv..

[4] Su S, Zaza P, Renken A (1994). Catalytic dehydrogenation of methanol to water-free formaldehyde. Chem. Eng. Technol..

[5] Guerrero-Ruiz A, Rodrigues-Ramos I, Fierro JLG (1991). Dehydrogenation of methanol to methyl formate over supported copper catalysts. Appl. Catal..

[6] Munnik P, de Jongh PE, de Jong KP (2015). Recent Developments in the Synthesis of Supported Catalysts. Chem. Rev..

[7] Chen LF, Guo PJ, Qiao MH (2008). Cu/SiO_2_ catalysts prepared by the ammonia-evaporation method: Texture, structure, and catalytic performance in hydrogenation of dimethyl oxalate to ethylene glycol. J. Catal..

[8] Matsuda T, Yogo K, Pantawong C, Kikuchi E (1995). Catalytic properties of copper-exchanged clays for the dehydrogenation of methanol to methyl formate. Appl. Catal. A: Gen..

[9] Sasaki K (2016). Synthesis of copper nanoparticles within the interlayer space of titania nanosheet transparent films. J. Mater. Chem. C.

[10] Frenkel M (1974). Surface Acidity of Montmorillonites. Clays Clay Miner..

[11] Guerreiro ED, Gorriz OF, Larsen G, Arrua LA (2000). Cu/SiO_2_ catalysts for methanol to methyl formate dehydrogenation. A comparative study using different preparation techniques. Appl. Catal. A: Gen..

[12] Minyukova TP (2002). Dehydrogenation of methanol over coppercontaining catalysts. Appl. Catal. A: Gen..

[13] Loiseau T, Férey G (2007). Crystalline oxyfluorinated open-framework compounds: Silicates, metal phosphates, metal fluorides and metal-organic frameworks (MOF). J. Fluorine Chem..

[14] Gui C-X (2014). Sandwichlike Magnesium Silicate/Reduced Graphene Oxide Nanocomposite for Enhanced Pb^2+^ and Methylene Blue Adsorption. ACS Appl. Mater. Interfaces.

[15] Hao S-M (2016). Hollow Manganese Silicate Nanotubes with Tunable Secondary Nanostructures as Excellent Fenton-Type Catalysts for Dye Decomposition at Ambient Temperature. Adv. Funct. Mater..

[16] Hao S-M, Yu M-Y, Zhang Y-J, Abdelkrim Y, Qu J (2019). Hierarchical mesoporous cobalt silicate architectures as high-performance sulfate-radical-based advanced oxidization catalysts. J. Colloid. Interf. Sci..

[17] Zhu Z-S (2020). Preforming abundant surface cobalt hydroxyl groups on low crystalline flowerlike Co_3_(Si_2_O_5_)_2_(OH)_2_ for enhancing catalytic degradation performances with a critical nonradical reaction. Appl. Catal. B: Environ..

[18] Niu X, Zhao T, Yuan F, Zhu Y (2015). Preparation of Hollow CuO@SiO_2_ Spheres and Its Catalytic Performances for the NO + CO and CO Oxidation. Sci. Rep..

[19] Lambert S, Cellier C, Ferauche F, Gaigneaux EM, Heinrichs B (2007). On the structure-sensitivity of 2-butanol dehydrogenation over Cu/SiO_2_ cogelled xerogel catalysts. Catal. Commun..

[20] Wang X, Zhuang J, Chen J, Zhou K, Li Y (2004). Thermally Stable Silicate Nanotubes. Angew. Chem. Int. Ed..

[21] Tang X-H, Wang J-Z, Li H-X (2004). Synthesis and characterization of nano-sized copper-silicate with mel structure. Stud. Sur. Sci. Catal..

[22] Narasimharao K, Ali TT, Bawaked S, Basahel S (2014). Effect of Si precursor on structural and catalytic properties of nanosize magnesium silicates. Appl. Catal. A: Gen..

[23] Prasadrao PRH, Kumar R, Ramaswamy AV, Ratnasamy P (1993). Synthesis and characterization of a crystalline vanadium silicate with MEL structure. Zeolites.

[24] Shen L, Zhong W, Wang H, Du Q, Yang YJ (2004). Preparation and characterization of SMA(SAN)/silica hybrids derived from water glass. Appl. Polym. Sci..

[25] Rakshe B, Ramaswamy V (1998). Synthesis and characterization of zirconium silicate molecular sieves of MEL type using two different zirconium sources. Stud. Sur. Sci. Catal..

[26] Sharma P, Chung W-J (2011). Synthesis of MEL type zeolite with different kinds of morphology for the recovery of 1-butanol from aqueous solution. Desalination.

[27] Toupance T, Kermarec M, Louis C (2000). Metal Particle Size in Silica-Supported Copper Catalysts. Influence of the Conditions of Preparation and of Thermal Pretreatments. J. Phys. Chem. B.

[28] Dong JP, Zou J, Long Y (2003). Synthesis and characterization of colloidal TBAsilicalite-2. Micro. Meso. Mater..

[29] Sing KSW, Williams RT (2004). Physisorption Hysteresis Loops and the Characterization of Nanoporous Materials. Adsorpt. Sci. Technol..

[30] Lyu L, Zhang L, Hu C (2015). Enhanced Fenton-like degradation of pharmaceuticals over framework copper species in copper-doped mesoporous silica microspheres. Chem. Eng. J..

[31] López-Suárez F (2009). Role of surface and lattice copper species in copper-containing (Mg/Sr)TiO_3_ perovskite catalysts for soot combustion. Appl. Catal. B Environ..

[32] Mosser C, Mosser A, Romeo M, Petit S, Decarreau A (1992). Natural and Synthetic Copper Phyllosilicates Studied by XPS. Clays Clay Miner..

[33] Kim K, Yi DK, Paik U (2017). CuO embedded silica nanoparticles for tungsten oxidation via a heterogeneous Fenton-like reaction. Microelectron. Eng..

[34] Karmouch R, Ross GG (2010). Super hydrophobic wind turbine blade surfaces obtained by a simple deposition of silica nanoparticles embedded in epoxy. Appl. Surf. Sci..

[35] Faqeeh AJ, Ali TT, Basahel SN, Narasimharao K (2018). Nanosized samarium modified Au-Ce0.5Zr0.5O_2_ catalysts for oxidation of benzyl alcohol. Mol. Catal..

[36] Basahel SN (2016). Physico-Chemical and Catalytic Properties of Mesoporous CuOZrO_2_ Catalysts. Catalysts.

[37] Narasimharao K, Ali TT (2013). Catalytic Oxidative Cracking of Propane over Nanosized Gold Supported Ce0.5Zr0.5O_2_ Catalysts. Catal. Lett..

[38] Arena F (2008). Solid-state interactions, adsorption sites and functionality of Cu-ZnO/ZrO_2_ catalysts in the CO_2_ hydrogenation to CH_3_OH. Appl. Catal. A: Gen..

[39] Tonner SP, Trimm DL, Wainwright MS, Cant NW (1984). Dehydrogenation of methanol to methyl formate over copper catalysts. Ind. Eng. Chem. Prod. Res. Dev..

[40] Shelepova EV, Ilina LY, Vedyagin AA (2017). Kinetic studies of methanol dehydrogenation. Part I: copper-silica catalysts. Reac. Kinet. Mech. Cat..

[41] Fridman V, Davydov AA (2000). Dehydrogenation of Cyclohexanol on Copper-Containing Catalysts: I. The Influence of the Oxidation State of Copper on the Activity of Copper Sites. J. Catal..

[42] Sato AG (2013). Effect of the ZrO_2_ phase on the structure and behavior of supported Cu catalysts for ethanol conversion. J. Catal..

